# Effects of Yeast (*Saccharomyces Cerevisiae*) Probiotics Supplementation on Bone Quality Characteristics in Young Japanese Quail (*Coturnix Japonica*): The Role of Sex on the Action of the Gut-Bone Axis

**DOI:** 10.3390/ani10030440

**Published:** 2020-03-05

**Authors:** Siemowit Muszyński, Piotr Dobrowolski, Kornel Kasperek, Sebastian Knaga, Małgorzata Kwiecień, Janine Donaldson, Mateusz Kutyła, Małgorzata Kapica, Ewa Tomaszewska

**Affiliations:** 1 Department of Biophysics, Faculty of Environmental Biology, University of Life Sciences in Lublin, Akademicka St. 13, 20-950 Lublin, Poland; 2 Department of Functional Anatomy and Cytobiology, Faculty of Biology and Biotechnology, Maria Curie-Sklodowska University, Akademicka St. 19, 20-033 Lublin, Poland; piotr.dobrowolski@umcs.lublin.pl; 3 Institute of Biological Basis of Animal Production, Faculty of Animal Sciences and Bioeconomy, University of Life Sciences in Lublin, Akademicka St. 13, 20-950 Lublin, Poland; kornel.kasperek@up.lublin.pl (K.K.); sebastian.knaga@up.lublin.pl (S.K.); 4 Institute of Animal Nutrition and Bromatology, Faculty of Animal Sciences and Bioeconomy, University of Life Sciences in Lublin, Akademicka St. 13, 20-950 Lublin, Poland; malgorzata.kwiecien@up.lublin.pl; 5 School of Physiology, Faculty of Health Sciences, University of the Witwatersrand, 7 York St., Parktown, Johannesburg 2193, South Africa; janine.donaldson@wits.ac.za; 6 Department of Industrial and Environmental Microbiology, Faculty of Biology and Biotechnology, Maria Curie-Sklodowska University, Akademicka St. 19, 20-033 Lublin, Poland; mkutya61@gmail.com; 7 Department of Animal Physiology, Faculty of Veterinary Medicine, University of Life Sciences in Lublin, Akademicka St. 12, 20-950 Lublin, Poland; malgorzata.kapica@up.lublin.pl

**Keywords:** quail, bone, yeast, sex, gut bone-axis

## Abstract

**Simple Summary:**

The gastrointestinal tract; as an important mediator of nutrients and elements; regulates bone health. In this study, we examined the effect of supplementation with yeast *Saccharomyces cerevisiae* on bone characteristics in young Japanese quails. We found that yeast probiotics, through their action on the gut-bone axis, have a positive effect on the structure of articular cartilage and microarchitecture of trabecular bone in young female quails. These data could provide useful information for further research into the supplementation with yeast probiotics aimed to reduce the risk bone fractures during the egg-laying period

**Abstract:**

The aim of the study was to investigate the changes in bone geometry, histological structure, and selected mechanical characteristics in young male and female Japanese quails supplemented with *Saccharomyces cerevisiae*. Quails were fed a basal diet containing no yeast or a basal diet supplemented with 1.5% (15 g per 1 kg of diet) of inactive *S. cerevisiae*, for a period of 42 days. *S. cerevisiae* inclusion had no effect on bone weight, length, and density, diaphysis geometry (cross-sectional area, wall thickness, moment of inertia) or on the mechanical strength (yield load, ultimate load, stiffness, Young’s modulus, yield stress, ultimate stress). Yeast supplementation improved the morphology of the articular cartilage both in male and female quails, as the total thickness of the articular cartilage was significantly increased. In trabecular bone, an increase in real bone volume and trabecular thickness was observed in females supplemented with *S. cerevisiae*, while in males the increase in trabecular number was accompanied by a reduction in trabecular thickness. The results of the present study demonstrate that *S. cerevisiae*, through a sex-dependent action on the gut-bone axis, improved the structure of articular cartilage and microarchitecture of trabecular bone. The positive effects of *S. cerevisiae* supplementation were more evident in female quails.

## 1. Introduction

The gastrointestinal tract, as an important mediator of nutrients and elements, has been known to regulate bone health through the absorption of bone minerals, including the essential macroelements, calcium and phosphorous. However, recent research performed on both mammals and avian species has suggested the existence of a “gut-bone axis”, which indicates that the digestive tract might have an even more complex role the in the maintenance of bone homeostasis [1]. While a comprehensive description of the gut-bone axis function has yet to be established, several mechanisms of action have been proposed, for which the gut structure plays an important role, demonstrating the pivotal importance of intestinal morphology and barrier function for enhancing skeletal health [2,3,4].

Several studies have shown positive effects of brewer’s yeast *Saccharomyces cerevisiae* probiotic on poultry performance, health, and feed efficiency [5,6]. It has been also reported that yeast probiotics have a stimulatory effect on bone development [7,8,9] which has been related, among others, to the phytase activity of *S. cerevisiae*, releasing minerals from phytic acid complex [10,11]. 

The Japanese quail (*Coturnix japonica*), the smallest of the avian species largely farmed for egg and meat production [12,13], is extensively used as an avian model animal in examining various physiological processes [14,15]. Very recently, we have demonstrated that supplementation with *S. cerevisiae* increased the size of the mucosal absorptive surface area in the duodenum of male quails, while female quails showed an increased absorptive surface area in the jejunum [16].

To our knowledge, no study examining the effects of yeast supplementation on the regulation of the gut-bone axis has been published. Therefore, in the present study, we tested the hypothesis that alterations in the intestine morphology can have an effect on the skeletal system. Specifically, we aimed to determine changes in bone geometry, histological structure and selected mechanical characteristics related to alterations in intestine morphology caused by (1) long term supplementation with yeast probiotic *S. cerevisiae* and (2) sex-dependent alterations in Japanese quail. 

## 2. Materials and Methods 

### 2.1. Ethical Approval

All experimental procedures used throughout this study were approved by the Local Ethics Committee on Animal Experimentation of the University of Life Sciences in Lublin, Poland (41/2016). 

### 2.2. Animals and Experimental Feeds

The study was carried out on male and female Japanese quail of standard S-33 line (n = 320), from the breading flock of the University of Life Sciences in Lublin. Bird management and care are described in detail in a previous publication [16]. Briefly, hatched quail chicks were randomly allocated to two dietary treatments (n = 160): fed either a basal diet containing no yeast (0 g yeast/kg as a control, the Y0 group) or a basal diet plus 1.5% (15 g yeast/kg) of *Saccharomyces cerevisiae* yeast inactivated by drying (the Y1 group) (Table 1). The *S. cerevisiae* supplementation dose was chosen on the preliminary study performed on male and female quails supplemented with 0.5%, 1.0%, 1.5%, 2.0%, 3.5% and 5.0% of S. *cerevisiae*, with the selection criterion of the highest S. *cerevisiae* dose with the fewest inflammatory lesions in the small intestine [16]. The examined dose range was selected on the basis of previously published trials where growing [17,18] or laying quails [19,20] were supplemented with S. *cerevisiae* doses ranging from 0.5% up to 16%. All feeds used in the experiment were prepared in an industrial feed mixing facility (Agropol, Motycz, Poland) in accordance with the arranged guidelines. The basal diet was formulated to meet or exceed nutrient requirements for quail [21]. Each of two experimental group was randomly subdivided into 8 subgroups (replicates), comprising of 20 birds each. After being separated at three weeks of age according to sex by a qualified technician, based on the colour of the plumage and through cloacal gland inspection, female and male quail in each group were transferred to separate cages. Therefore, a 2 × 2 factorial arrangement was employed; *S. cerevisiae* inclusion (0 or 15 g/kg of feed) and quails’ sex (male or female) as factors with 8 replicate cages per each experimental group (32 cages in total). Each replication cage contained 10 birds with the expectation of 7 cages containing 9 birds, because it was not possible to select the target number of individuals of one sex in these replications. The experiment lasted until 6 weeks of age. At the end of the experiment, 8 birds were randomly selected from each group (one from cage replicate), fasted for 12 h, individually weighed and sacrificed by decapitation after mechanical stunning (32 birds in total, 8 individuals from each of the four experimental groups). Both tibiae were carefully dissected, cleaned from adhering tissues and kept frozen at −25 °C until further examination. 

### 2.3. Bone analysis

Before all analyses, the frozen bones were thawed overnight at 5 °C. A three-point bending test of the right tibiae was performed on a universal testing machine (Zwick Z010, Zwick/Roell GmbH & Co, Ulm, Germany) to assess the mechanical properties of the bones. Prior to the analyses, tibia weight, length, and Seedor index (bone weight to length ratio, which can be used to describe the degree of bone mineralization) were determined. During the bending test, the load was applied at the middle point of the bone diaphysis in the cranial-caudal plane, with a loading rate of 10 mm/min-1 until fracture [23]. Structural properties of the bone diaphysis (yield load, ultimate load, and stiffness) were read from recorded force-displacement curves using Origin 2016 software (OriginLab, Northampton, MA, USA). Next, the measurement of bone volumetric density was performed with a helium gas pycnometer, as previously described [24]. Finally, the bones were defatted in a 1:1 mixture of chloroform and methanol for 24 h, weighed and ashed in a muffle furnace at 500 °C for 24 h to determine bone ash percentage. The percentage ash was determined relative to dry weight of the tibia [25].

Left tibiae were cut in the midpoint of the bone diaphysis with a diamond bandsaw (MBS 240/E, Proxxon GmbH, Foehren, Germany). Geometric properties of the bone diaphysis (cross-sectional area and mean relative wall thickness), as well as geometric indices of distribution of cortical bone mass (cross-sectional moment of inertia, radius of gyration) were calculated on the basis of measurements of external and internal diameters of the bone diaphysis cross-section, assuming its elliptical shape [26]. The data collected during the three-point bending tests and measurements of bone diaphysis cross-sectional geometry were used for the calculations of bone material properties (yield stress, ultimate stress, Young’s modulus) using appropriate beam-theory equations [27].

### 2.4. Bone Histomorphometry

After the geometric measurements, the proximal ends of the left tibiae were cut off and fixed in 4% buffered formaldehyde. Formalin-fixed samples were dehydrated and cleared with Ottix Plus and Ottix Shaper solvent substitutes (DiaPath, Martinengo, Italy) and then embedded in paraffin. Four, 4-μm thick, semi-serial cuts of the sagittal sections of the lateral condyle were sectioned with a microtome and placed on one microscopic slide. The sections were stained with Safranine O to evaluate the morphology of the articular cartilage and for the assessment of the microarchitecture of trabecular bone [28]. Stained sections were observed in normal light by light microscopy (CX43, Olympus, Tokyo, Japan). Eight microscopic fields (two from each cut) from each slide were recorded using a high-resolution CDD camera (SC50, Olympus, Tokyo, Japan). The analysis of the collected images of articular cartilage was performed with the use of Olympus cellSens software (Olympus, Tokyo, Japan). On each image the total thickness, as well as the thickness of the main zones: horizontal zone (the zone I), transitional zone (the zone II), and radial zone (the zone III) were measured at four sites along the cartilage. The trabecular bone microarchitecture was assessed on the basis of measurements performed using an appropriate tool in ImageJ software (Wayne Rasband, National Institute of Mental Health, Bethesda, MD, USA). The following parameters were determined: bone volume (BV/TV), mean trabecular thickness (Tb.Th mean), maximal trabecular thickness (Tb.Th mean), mean trabecular space (Tb.Sp mean), maximal trabecular space (Tb.Sp max), and trabecular number (Tb.N) [29].

### 2.5. Statistical Analysis

The data were analyzed using a two-way analysis of variance (2 × 2 factorial design), with the model including *S. cerevisiae* supplementation (0 or 15 g/kg of feed) and with quails’ sex as the main factors; interactions were analyzed using a general linear model (GML). Duncan’s multiple range test was employed to determine means and differences among treatments. Significant differences were determined for *p* < 0.05.

## 3. Results

### 3.1. Bone Morphology, Geometry, and Mechanical Traits

Tibia morphological properties and densitometric data are presented in Table 2. Female quails had significantly heavier bones than males, irrespective of *S. cerevisiae* supplementation (*p* < 0.05). Similarly, there was a significant effect of sex on bone volumetric density (*p* < 0.001) and bone ash percentage (*p* < 0.001), both of which were greater in females. There was no effect of sex or yeast addition on tibia length and Seedor index. Cross-sectional moment of inertia (CSMI) and radius of gyration of bone diaphysis cross-section were dependent on quails’ sex, with greater values observed in females (*p* < 0.05 in both cases). 

The results of the analysis of tibia biomechanical properties are summarized in Table 3. Ultimate load was significantly affected by quail sex (*p* < 0.001), with higher values recorded for female quail. There was no effect of quails’ sex or *S. cerevisiae* supplementation on other bone structural (yield load, stiffness) and material properties (Young’s modulus, yield stress, and ultimate stress).

### 3.2. Trabecular Bone Microarchitecture and Articular Cartilage Histomorphometry

**Figure 1:**
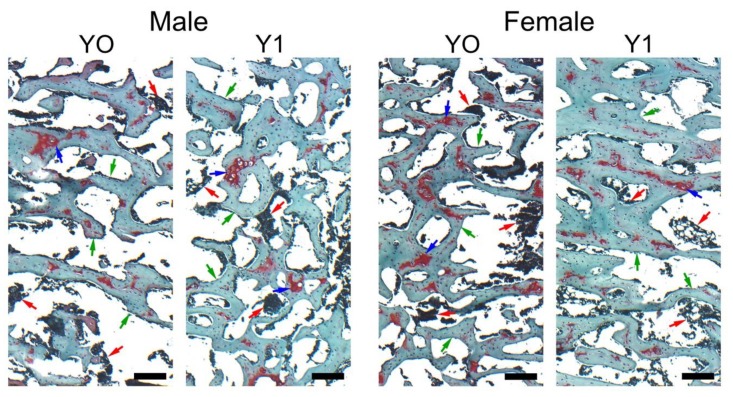
Representative images of Safranine O staining carried out on formaldehyde-fixed sections of trabecular bone in the proximal section of the tibia from representative 6-week old control (Y0) and yeast-fed (Y1) Japanese quail. Green arrows indicate trabeculae, red arrows indicate bone marrow, blue arrows indicate areas of reduced mineralization of newly formed trabeculae. All the scale bars represent 100 µm.

Table 4 shows the results of the analysis of trabecular bone microarchitecture and articular cartilage histomorphometry. The effect of yeast supplementation was sex-dependent, as indicated by numerous interactions. Representative microscopic images of trabecular bone of quails un-supplemented and supplemented with *S. cerevisiae* stained with Safranine O are presented in Figure 1. The lowest bone volume density (BV/TV) was observed in tibiae of female quail from the control group, which increased after yeast supplementation (*p* < 0.001) (Figure 1). Mean and maximum trabecular thickness increased in females and decreased in males supplemented with *S. cerevisiae* (*p* < 0.001 in both cases). On the other hand, trabecular number increased only in the tibiae of yeast-supplemented male quails. There was no effect of quail sex or *S. cerevisiae* inclusion on mean and maximum trabecular space.

In tibia articular cartilage, total thickness of articular cartilage as well as the zone I thickness increased in both male and female quail after *S. cerevisiae* inclusion, but to a greater extent in males (*p* < 0.001 and *p* < 0.05, respectively; Table 4). An increase in the thickness of zone II after yeast supplementation was observed only in female quail (*p* < 0.001). Female quail from the control group displayed the thickest zone III articular cartilage, while the males from the control group had the thinnest zone III articular cartilage. In yeast supplemented groups, the thickness of zone III was increased in males and decreased in females (*p* < 0.001).

## 4. Discussion

The current study was designed to evaluate the effects of yeast supplementation on bone structure and mechanical properties in male and female Japanese quail. In poultry, skeletal development is influenced by numerous factors, including nutrition and health status [30]. In a review by Mcbabe et al. [31], it was recommended that the comprehensive analysis of probiotic regulation of bone health should address the following aspects: selection of an appropriate animal model, age and sex of the animals used, analysis of bone mineralization, bone mechanical testing and the analysis of trabecular bone. In our study, we made use of the Japanese quail as an animal model. This species has been routinely used as an animal model for in vivo studies on bone development across various disciplines within the biological sciences for decades [32,33,34], which makes it an optimal model for a study on the probiotic regulation of bone health. Our study was performed on both male and female quail, and to avoid the effects of egg production on bone homeostasis in hens (i.e., formation of medullary bone), our study was performed on young, rapidly growing quail that did not lay eggs. The traits analyzed in our study included the assessment of bone mineralization and morphological properties, detailed analysis of the mechanical properties of bone mid-diaphysis and assessment of histomorphometry of the articular cartilage and microarchitecture of trabecular bone. The analyses conducted in the present study provided us with detailed information concerning the effect of a yeast probiotic on bone development in a poultry animal model.

Female quail are generally heavier than males [35], and the same was observed in our study [16]. As a result, the bones of the female quail were heavier, more dense (as evident in the Seedor index and bone volumetric density) and more mineralized (bone ash percentage) than those of the males. This result is in agreement with that reported previously by Nishimura et al. [36], who showed that female quail had higher bone densities compared to those noted for males. However, no effect of sex on tibia length was observed, which was also previously reported by Nishimura et al. [36].

Bone density measurements have been widely used to assess bone mechanical endurance [37]. However, the quality of the collagen within the bone and the bone diaphysis geometric indices also provide important information concerning bone strength [33,34]. In bending, bone load-carrying capacity and the bone’s resistance to an imposed bending load, directly depends on the area moment of inertia about the bending axis for a specific cross-section of the bone diaphysis, for a particular bone [26]. Thus the cross-sectional moment of inertia is an important geometric index of bone rigidity [38].

The sex differences in bone mineralization, volumetric density and cross sectional moment of inertia indicate positive adaptations in bone structure towards supporting the increased body mass of female quail. Bone quality (bone mineralization), as well as bone diaphysis spatial mass distribution (cross-sectional moment of inertia and radius of gyration), are critical in the bone’s ability to counteract the loads associated with body weight [39]. In bending, ultimate stress is the measure that describes the effect of diaphysis cross-sectional geometry on bending strength and is directly proportional to the ultimate load a bone can sustain during bending. Therefore, although there was no change in bone length or weight, various physiological mechanisms allowed the bones to adapt their structure (more rapid periosteal expansion during growth) in order to withstand greater loads (represented in performed three-point bending test by ultimate load) and to maintain whole bone strength (yield and ultimate stress), despite the heavier body mass of the female quail. 

Probiotics can beneficially influence bones by stimulating the action of vitamins which participate in the metabolism of calcium [11]. It was reported that the addition of *S. cerevisiae* to feed increases the concentration of calcitriol receptors [40]. Calcitriol, the active form of vitamin D, increases calcium absorption from the intestine by the enterocytes, through an increase in the production of calcium transport proteins [41]. Increased levels of serum calcium, resulting from calcitriol promoted intestinal uptake, can counterbalance bone calcium losses resulting from hormonal stimulation of osteoclasts [42]. However, this effect of *S. cerevisiae* supplementation was probably not observed in our study since there was no increase in calcium serum concentrations observed in the groups supplemented with *S. cerevisiae* [16]. Similarly, *S. cerevisiae* supplementation had no effect on tibia ash content and bone ash percentage, which have been widely used as predictors of calcium and phosphorus content in bone. The lack of changes observed in bone ash content following supplementation with the yeast probiotic is in agreement with previous studies, where tibia ash content remained unchanged in broilers fed a normal [43] or low phosphorus diet [7] supplemented with *S. cerevisiae* for 42 days. Angel et al. [44] and Houshmand et al. [8] showed that feeding broilers diets containing lowered levels of calcium resulted in lower tibia ash, but the addition of a probiotic to the low-calcium diets overcame this negative effect. Sacakli et al. [9] showed that yeast supplementation (1, 3, and 5%) prevented the reduction in tibia ash content in chickens fed diets with low vitamin and trace mineral content.

In our previous work, we have shown that probiotic supplementation (strains of four bacterial and one yeast culture) may improve bone geometry and increase bone mechanical strength in poultry [45]. In the present study, *S. cerevisiae* supplementation had no effect on any of the tibia morphological or biomechanical parameters measured. Due to the limited number of previous studies similar to ours, a detailed comparison with previous results was not possible. Moreover, none of the previous studies have examined the effect of *S. cerevisiae* on bone morphology and mechanical performance to such an extent. In broiler chickens fed a low-calcium diet supplemented with yeast (0.2%), an increase in tibia length and weight was observed, but these changes did not influence the Seedor index or bone mechanical strength [8]. Ambiguous results were also obtained by Suzer et al. [42], where broiler chickens fed diets containing 0.1%, 0.2%, and 0.4% *S. cerevisiae* displayed increased tibia weight, length, and cortical area as well as a reduction in bone breaking strength, all of which were dose-dependent. On the other hand, Plavnik and Scott [46] reported that when complete broilers’ diet was supplemented with 2.5 and 5.0% brewer’s dried yeast, improvements in leg weaknesses were observed: chickens were characterized with stronger bones and lower incidence of tibial dyschondroplasia.

We have not yet come across any other studies in which the effects of yeast on trabecular bone microarchitecture or articular cartilage histomorphometry were analyzed. In the current study the effects of yeast supplementation on trabecular bone microarchitecture were sex-dependent. Yeast probiotic supplementation enhanced bone development and increased bone mineral density at the proximal epiphysis in female quail, as evidenced by the enhanced thickness of trabeculae which resulted in higher BV/TV (real bone volume). Additionally, a reduced number of red-stained areas, as seen in Figure 1, suggest the improved mineralization of trabeculae in the supplemented group. In males no change in real bone volume was observed, as the increase in trabecular number was accompanied by a reduction in trabecular thickness. Taken together, the results of the current study indicate that bone formation in the tibia increases in response to *S. cerevisiae* supplementation, leading to increased trabecular bone volume in female quail. This sex-dependent effect of bacteria probiotic supplementation was previously reported in mice, where *Lactobacillus reuteri* increased trabecular bone formation only in males, which was accompanied by a decrease in intestinal inflammation, showing that the action of the gut-bone axis can indeed be sex-dependent [31]. 

Yeast supplementation improved the morphology of the articular cartilage. These improvements can have functional consequences, as the physical properties of articular cartilage are determined by the thickness of the various cartilage zones [47]. For example, the thickening of the horizontal zone (I) can enhance the elasticity of the articular cartilage and improve the load distribution in the joint, thus protecting it against excessive degradation in heavier females. Similarly, the increase in thickness of the transitional (II) zone in female quail can be equally beneficial, since it improves the transfer of the load through the cartilage.

*S. cerevisiae* has the ability to biodegrade phytate, which enhances not only the bioavailability of phosphorus or calcium but also that of iron, zinc, and magnesium from phytic acid complex [10,11]. Copper, zinc, manganese and magnesium are essential cofactors for enzymes involved in collagen synthesis and other bone matrix constituents that are required in bone and cartilage formation processes [48,49]. Thus, this enhanced mineral bioavailability may be directly related to the observed improvements in trabecular bone microarchitecture and articular cartilage histomorphometry, as has been reported in numerous previous studies on poultry, by our laboratory [50,51,52,53].

Tomaszewska et al. [16] showed that the intestinal mucosa of quail fed *S. cerevisiae* develops in a sex-dependent manner [16]. Supplementation with *S. cerevisiae* caused significant positive effects on the morphology of the small intestine, increasing the size of the mucosal absorptive surface area in the duodenum in male quail, while the absorptive surface area in the jejunum increased in female quail [16]. Zinc is primarily absorbed in the duodenum and proximal jejunum and the absorption of zinc is impaired by phytate [54]. Magnesium ions are absorbed primarily in the jejunum and ileum, with both passive and active transport processes implicated [55]. Finally, copper and iron absorption occurs primarily in the duodenum [55]. However, intestine surface area cannot be the only factor associated with the action of yeast in improving the absorption of various minerals, since it was also effective in males, in whom an increase in absorptive surface area in the duodenum was not observed.

Together, the results of the present study demonstrate that the influence of *S. cerevisiae* on bone health revealed itself only in trabecular bone and articular cartilage. The possibility that alterations in mineral absorption influenced our findings is likely, mainly for the reason that trabecular bone volume fraction differed between un-supplemented and supplemented groups, whilst tibia length was similar across groups. What is more, our results are in agreement with previous studies performed on other animal models, mainly rats, where it has been postulated that effects of probiotic supplementation are more visible in the more metabolically active trabecular bone, than in cortical bone [1,2]. However, in our study the effects of the yeast probiotic on bone health in young Japanese quails were sex-dependent. In our opinion, this is the most important outcome of our study.

## 5. Conclusions

In our study, conducted on the Japanese quail model, we have shown many positive effects of yeast supplementation on the bones of the quail, especially in females. Furthermore, treatment with *S. cerevisiae* had a more significant effect on trabecular bone morphology than on whole-bone strength. This observation may have some potential applications. Yeast probiotics improve trabecular bone and articular cartilage structure in female Japanese quails at the beginning of the egg-laying period. This could suggest that in the later life during the egg-laying period the bone loss rate could be reduced, leading to lower risk of bone fracture. However, given the fact that *S. cerevisiae* may interact with sex hormones, further research on mature quails are required to confirm this hypothesis.

## Figures and Tables

**Table 1:** Composition and nutritive value of feed mixtures for Japanese quail.

Ingredient (g/kg)	The Y0 group ^1^	The Y1 group ^1^
Maize	200	200
Wheat	263.55	265.35
Soybean meal, 48% CP ^b^	330	317
Yeast (*S. cerevisiae*)	0	15
Potato protein concentrate	80	80
Skim milk powder	20	20
Protein-fat concentrate	20	20
Monocalcium phosphate	19	19
Limestone	14	14
Sodium bicarbonate	0.6	0.6
Premix ^a^	5	5
Vegetable oil	40	36
DL-methionine 99%	2.5	2.5
L-lysine HCl	0.4	0.6
L-threonine 99%	1.2	1.2
Sodium chloride	3.75	3.75
Content in 1 kg of mixture		
^b^ AMEn, MJ	12.565	12.567
^c^ Crude protein, g/kg	280	280
^c^ Crude fat, g/kg	61.4	57.8
^c^ Crude fibre, g/kg	27.1	27.6
^c^ Lysine, g/kg	16.7	16.7
^c^ Methionine, g/kg	6.95	6.97
^c^ Methionine + Cysteine, g/kg	11.5	11.5
^c^ Tryptophan, g/kg	3.53	4.03
^c^ Threonine, g/kg	9.56	9.54
^c^ Ca, g/kg	11.3	11.3
^c^ Bioavailable P, g/kg	5.43	5.43
^c^ Total P, g/kg	8.29	8.33
^d^ Ca/bioavailable P	2.073	2.075
^c^ Na, g/kg	1.70	1.75

^a^ The premix provided in 1 kg of diet: Mn 60 mg; I 1 mg; Fe 54 mg; Cu 11 mg; Se 0.2 mg; vitamin A (retinol) 3.0 mg; vitamin D3 (cholecalciferol) 0.06 mg; vitamin E (alpha-tocopherol) 20 mg; vitamin K3 (menadione) 2 mg; vitamin B1 (thiamine) 1.5 mg; vit. B2 (riboflavin) 4.5 mg; vitamin B6 (pyridoxine) 3 mg; vitamin B12 (cyanocobalamin) 0.015 mg; biotin 0.1 mg; folic acid 0.8 mg; nicotinic acid 20 mg; pantothenic acid 12 mg; choline 300 mg. ^b^ CP – crude protein; AMEn – metabolizable energy at zero nitrogen balance, as calculated by Fisher and McNab [22] equations. ^c^ analyzed values. ^d^ calculated values. ^1^ Y0 – control group fed without yeast addition. ^1^ Y1 – group fed 1.5% of yeast (*S. cerevisiae).*

**Table 2:** Effect of dietary supplementation with *S. cerevisiae* (1.5%) on morphological parameters of tibia from male and female quail at 42 days of age.

Parameter	Y0 ^1^		Y1 ^1^		SEM	Influence		
	Male	Female	Male	Female		Yeast	Sex	Y x S ^2^
Morphological properties								
Tibia mass, g	1.17	1.31	1.25	1.30	0.03	0.281	0.012	0.176
Tibia length, mm	46.9	46.6	46.3	45.3	0.6	0.095	0.279	0.539
The Seedor index, mg/mm	24.9	28.0	27.1	28.8	0.7	0.061	0.004	0.348
Volumetric density, cm^3^	1.65	1.82	1.62	1.84	0.02	0.999	<0.001	0.209
Ash, %	42.3	48.9	41.8	48.3	0.4	0.249	<0.001	0.859
Bone diaphysis geometry								
Cross-sectional area, mm^2^	3.14	3.09	3.03	3.21	0.11	0.940	0.553	0.265
MRWT, --	1.05	1.00	1.09	1.32	0.09	0.051	0.230	0.135
CSMI, mm^4^	1.96	1.99	1.60	2.14	0.12	0.513	0.012	0.062
Index of gyration, mm	0.766	0.790	0.729	0.786	0.020	0.303	0.048	0.412

Values represent means of 8 replicate pens with 1 bird from each pen. SEM - standard error of the means. MRWT – mean relative wall thickness; CSMI – cross-sectional moment of inertia. ^1^ Y0 – control group fed without yeast addition. ^1^ Y1 – group fed 1.5% of yeast (*S. cerevisiae).* ^2^ Y x S – (yeast) x (sex) interaction.

**Table 3:** Effect of dietary supplementation with *S. cerevisiae* (1.5%) on biomechanical parameters of tibia in male and female quail at 42 days of age.

Parameter	Y0 ^1^		Y1 ^1^		SEM	Influence		
	Male	Female	Male	Female		Yeast	Sex	Y x S ^2^
Structural properties								
Yield load, N	33.5	32.1	29.9	33.3	2	0.517	0.613	0.211
Ultimate load, N	35.6	38.4	34.1	41.1	1.1	0.605	<0.001	0.066
Stiffness, N/mm	54.9	54.4	55.1	54.0	5	0.978	0.865	0.943
Material properties								
Young’s modulus, GPa	5.46	5.36	6.28	4.67	0.54	0.896	0.123	0.173
Yield stress, MPa	112	105	109	100	8	0.560	0.284	0.796
Ultimate stress, MPa	130	129	136	2126	8	0.811	0.444	0.576

Values represent means of 8 replicate pens with 1 bird from each pen. SEM - standard error of the means. MRWT – mean relative wall thickness; CSMI – cross-sectional moment of inertia. ^1^ Y0 – control group fed without yeast addition. ^1^ Y1 – group fed 1.5% of yeast (*S. cerevisiae).* ^2^ Y x S – (yeast) x (sex) interaction.

**Table 4:** Effect of dietary supplementation with *S. cerevisiae* (1.5%) on histomorphometrical parameters of the proximal physis of tibia from male and female quail at 42 days of age.

Parameter	Y0 ^1^		Y1 ^1^		SEM	Influence		
	Male	Female	Male	Female		Yeast	Sex	Y x S ^2^
Trabecular bone microarchitecture								
BV/TV, %	24.7 ^b^	19.2 ^a^	23.1 ^b^	24.9 ^b^	1.0	0.041	0.072	<0.001
Tb.Th mean, μm	42.0 ^b^	26.8 ^a^	30.0 ^a^	40.6 ^b^	2.6	0.755	0.412	<0.001
Tb.Th max, μm	128 ^c^	75.8 ^a^	91.3 ^ab^	112b ^c^	7.7	0.979	0.048	<0.001
Tb.Sp mean, μm	150	137	119	144	8.3	0.176	0.444	0.027
Tb.Sp max, μm	343	323	357	334	29	0.667	0.450	0.972
Trabecular number, /mm	6.28 ^a^	7.24 ^ab^	8.10 ^b^	6.14 ^a^	0.35	0.315	0.161	<0.001
Articular cartilage histomorphometry								
Total thickness, μm	549 ^a^	550 ^a^	771 ^c^	633 ^b^	13	<0.001	<0.001	<0.001
Zone I thickness, μm	20.9 ^a^	16.4 ^a^	37.6 ^c^	27.9 ^b^	1.2	<0.001	<0.001	0.034
Zone II thickness, μm	151 ^b^	100 ^a^	171 ^bc^	184 ^c^	8	<0.001	0.016	<0.001
Zone III thickness, μm	179 ^a^	313 ^c^	258 ^b^	266 ^b^	6	0.006	<0.001	<0.001

Values represent means of 8 replicate pens with 1 bird from each pen. a, b, c - mean values in rows with different letters differ significantly at *p* < 0.05. SEM - standard error of the means. BV/TV – relative bone volume; Tb.Th – trabecular thickness; Tb.Sp – trabecular separation; Tb.N – trabecular number. ^1^ Y0 – control group fed without yeast addition. ^1^ Y1 – group fed 1.5% of yeast (*S. cerevisiae).* ^2^ Y x S – (yeast) × (sex) interaction.
